# Do Fungi Undergo Apoptosis-Like Programmed Cell Death?

**DOI:** 10.1128/mBio.00948-18

**Published:** 2018-07-31

**Authors:** J. Marie Hardwick

**Affiliations:** a Department of Molecular Microbiology and Immunology, Johns Hopkins University, Bloomberg School of Public Health, Baltimore, Maryland, USA; Geisel School of Medicine at Dartmouth; Tel Aviv University

**Keywords:** apoptosis, cell death, cell-autonomous, fungi, mycoptosis, necroptosis, programmed cell death, yeast, pathogenic fungi, MLKL

## Abstract

This question of whether fungi undergo apoptosis-like programmed cell death can be separated into two questions. One question is about applying the term "apoptosis" to fungi, and the other is a more challenging question of whether fungi have evolved mechanisms that inflict self-injury. The answers to both questions depend on the definitions applied to “apoptosis” and “programmed cell death.” Considering how these and other cell death terms originated and are currently defined for animals, some confusion arises when the terms are applied to fungi. While it is difficult to defend the concept of fungal apoptosis, the more interesting issue is whether fungi will eventually be found to encode programmed or extemporaneous self-destructive processes, as suggested by intriguing new findings.

## Abstract (precis)

This question of whether fungi undergo apoptosis-like programmed cell death can be separated into two questions. One question is about applying the term “apoptosis” to fungi, and the other is a more challenging question of whether fungi have evolved mechanisms that inflict self-injury.

## PERSPECTIVE

The possibility that fungi, bacteria, parasites, and other microorganisms undergo programmed cell death (PCD) has been an emerging issue for many years (1, 2). Nowhere is this debate more intense than for fungi, but no consensus has been reached. Resolution currently rests on the fulfillment of divergent nonstandardized criteria. Aside from the inherent value and intrigue of new science, interest in this topic is spurred by the prospects for novel therapeutics and possible curtailment of the animal and plant devastations ascribed to fungi, such as annihilation of amphibian species and European and North American trees (3, 4). Taking the reverse perspective, knowledge about fungal cell death might help guide the preservation of diverse fungal species as world food resources, medicines, and the critical terrestrial ecosystems that we depend on (5–7). However, the relevant intracellular molecular events that occur in microbes as they die are not known.

## DERIVATION OF THE TERM “PROGRAMMED CELL DEATH”

Initiating discussions about fungal cell death is challenged by the lack of a vocabulary with generally agreed-upon definitions. The definition of “programmed cell death” also holds layers of complexity. The concept of physiological cell death arose in the 19th and 20th centuries when investigators observed the systematic disappearance of cells in various developing animal models (8). With the discovery of lysosomes in 1955 (9), some researchers in the cell death field became occupied with the idea that leakage of hydrolases from these “suicide bags” was to blame, while others argued that unleashed hydrolases were a consequence rather than a cause of cell death (10). These ideas have been revisited and extended more recently both in human disease (11) and in yeast cell death (12).

In pursuit of the components that control developmental cell death, Richard Lockshin and his PhD adviser Carroll Williams at Harvard published a series of papers in 1964 to 1965 applying the term “programmed cell death” to describe the dying process of larval abdominal muscle cells in newly emerged adult American silkmoths (13–17). Lockshin explains that this word choice was a metaphor, “a felicitous turn of phrase designed to exploit the trendiness of the then-nascent computer era”—hence “programmed,” analogous to developmental programs (18). By eliminating some potential causes of cell death, Lockshin and Williams also conceptualized the death as a "cell-autonomous process," meaning that the cell destined to die is capable of facilitating its own death (with or without triggers from neighboring cells) (13).

The accumulation of knowledge in the subsequent two to three decades from studies of cancer, virus infections, and, most notably, the worm Caenorhabditis elegans finally produced unequivocal evidence of an evolutionarily conserved, genetically encoded program of self-elimination (19–23). Identification of cell death genes in worms by the use of genetics (Nobel Prize, 2002), combined with the disease relevance revealed by mammalian models, was soon solidified by *in vitro* reconstitutions and crystal structures (24–26). Perhaps most salient was the discovery of worm *ced-3*, a gene required for developmental elimination of cells (19). Its sequence homology with the gene encoding the mammalian protease that activates proinflammatory cytokine interleukin-1β (IL-1β) (IL-1β-converting enzyme [ICE]), now designated caspase-1 (mediator of nonapoptotic death by pyroptosis), revealed that worm CED-3 likely causes cell-autonomous programmed cell death via its proteolytic activity (27, 28). The trail quickly led to identification of mammalian caspase-3, the mediator of mammalian apoptosis (29). Another key worm homologue, human BCL-2 (C. elegans CED-9), was a novel oncogene of unknown function (30–32) with viral equivalents (E1B-19k and BHRF1) (21, 33). BCL-2 became the first known antiapoptotic protein and the first apoptosis designer drug target and was approved by the FDA for cancer therapy in 2016 (34, 35). A large field of human/animal programmed cell death research sprang up in the 1990s and captured all the major meeting venues and continues through the present. This field focuses on metazoan cell death, leaving room for emergence of a new field of fungal cell death marked by the establishment in 2002 of IMYA (International Meeting on Yeast Apoptosis [a name change is under consideration]), which continues to grow (36, 37).

Programmed cell death, subsequently dubbed "PCD," is currently applied to all types of physiological cell death, encompassing both developmental and nondevelopmental processes required to sustain health (38). A newer term, “regulated cell death” (RCD), has gained usage as recommended by the Nomenclature Committee on Cell Death (NCCD) (39). RCD includes PCD and further encompasses nonphysiological/pathological cell death, though these terms are often used interchangeably, in part because the same death mechanisms that occur physiologically can also occur in disease states or be induced therapeutically.

Given that the terms PCD and RCD were developed to describe cell death in metazoans, some confusion arises when the terms are applied to single-cell species (though fungi typically form multicellular structures). Despite advice against the coining of neologisms from the NCCD and a new parallel guideline specifically intended for yeast that pushes the concept of yeast RCD (40), new nomenclature might be useful (e.g., to conduct literature searches on fungal cell death versus infected host cell death). The term “phenoptosis” (programmed death of an entire organism, multicellular or unicellular) (41, 42) is an appealing descriptor, or perhaps another derivative of the word apoptosis such as “fungitosis” or “mycoptosis” to specify fungal death should be adopted. First, consider how the existing terms arose and why they are thus not readily applicable to microbes.

## CRITERIA FOR CLASSIFICATION AS “PROGRAMMED CELL DEATH”

**FIG 1:**
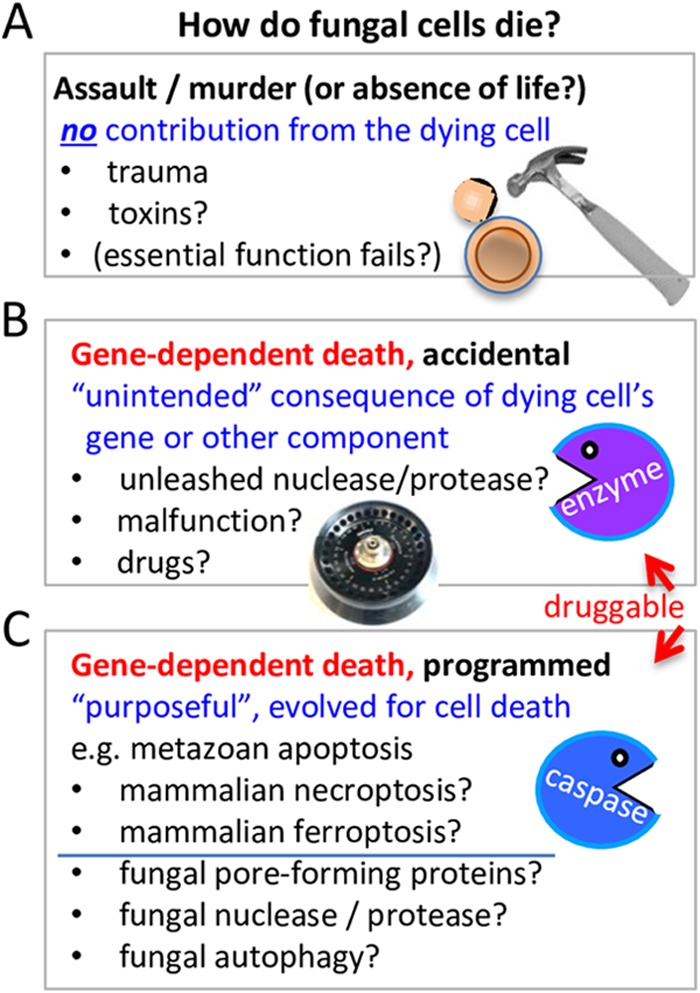
Three broad categories of cell death can be distinguished based on the level of participation by the dying cell. (A) Cell death that occurs without any contributions from the cell that dies is analogous to death by assault or murder (i.e., death is not dependent on a gene or other component originating in the cell destined to die), or, potentially, a cell could expire (like a car running out gas), though enthusiasm for the latter idea has waned in recent years. (B and C) A gene product or other component of the dying cell can contribute to its demise (gene-dependent, cell-autonomous death) in two conceptually distinct processes: accidental/extemporaneous cell death occurring via mechanisms not selected by evolution (B) and programmed cell death occurring via mechanisms selected during evolution (C). Cell death in all three categories (A to C) can be induced by conditions/factors (hammer, toxin, death receptor ligand, growth factor withdrawal, irradiation, sunlight, etc.) external to the cell that dies, while only cell death in categories B and C can also be triggered by events inside the cell, for example, events analogous to those involving the centrifuge rotor (B) or inherent errors in replication (C). These definitions differ somewhat from the NCCD definitions (39).

When one bacterial cell releases a toxin that kills a different bacterial cell, should this be considered programmed cell death? It depends. Few would doubt that bacterial killer toxins were selected during evolution for the ability to kill their target cells and in this way could be considered to represent a type of nonautonomous cell death “program.” However, the death mechanisms involved do not necessarily qualify as “programmed cell death” unless additional criteria are met. The burden of proof requires the dying cell to contribute actively to its own death (i.e., in a cell-autonomous manner). If the dying cell makes no contribution to its own death, this death can be considered equivalent to murder—the cell was simply hammered by forces beyond its control (Fig. 1A). An analogous example from mammals might be perforin, which is released from cytotoxic lymphocytes and forms pores in target cell membranes (43). (The NCCD unfortunately refers to death by assault as “accidental” cell death [ACD] [39].) A possible variant of death by assault is the complete absence of any death mechanisms or processes; in such a case, death is defined simply as the absence of life (life simply stops, like a car that runs out of gas). This way of thinking was once prominent but has faded with the accumulation of knowledge about death (Fig. 1A). However, even if there is compelling evidence that a dying cell is capable of contributing to its own death, this is still not sufficient to qualify as “programmed cell death.”

There is one more hurdle. Theoretically, a microbial toxin could trigger two conceptually distinct types of events in the target cell destined to die; both events require contributions by the dying cell, but only one represents programmed cell death. The distinction between them depends on the answer to the following question: did a gene product or other component originating from the dying cell "purposely" contribute to its own death? If yes, the death fits the long-held definition of “programmed cell death” as currently interpreted (39).

## PROGRAMMED VERSUS GENE-DEPENDENT CELL (OR CENTRIFUGE) DEATH

To illustrate that final distinction between programmed and nonprogrammed cell death, take the case of the laboratory centrifuge. Let the centrifuge represent the dying cell, and the centrifuge rotor represent the death-promoting gene product encoded by the dying cell. If the rotor fractures while spinning and wrecks the centrifuge beyond repair, this would be analogous to a cell’s protein actively contributing to the cell's own death. In this case, the centrifuge/cell-killing function of the rotor/protein (e.g., an unleashed protease or nuclease) was not a feature of the rotor/protein design. Cell death analogous to this centrifuge example can be classified as cell-autonomous and gene-dependent death (i.e., a gene or other component originating from the dying cell helps promote self-elimination). Here I refer to this type of cell death as accidental or extemporaneous (metaphorically, “unintended cell death”) (Fig. 1B). However, if the centrifuge rotor had been designed by its engineers to destroy the centrifuge when its time was up, this would be considered programmed death (metaphorically, “deliberate cell death”) (Fig. 1C). Thus, cell-autonomous mechanisms that evolved for cell death, or that can be reasonably anticipated as such, are generally considered to be programmed death mechanisms. Note, however, that the rotor/protein has another “day job” function in the centrifuge/healthy cell.

Importantly, both types of gene-dependent death (programmed and nonprogrammed) are potentially druggable; thus, the question of their evolutionary origins seems less important. Furthermore, distinguishing between accidental/unintended death and deliberate/programmed death may not be feasible experimentally, even in animals. Therefore, a more readily testable definition would be useful. We ask whether the death of a cell is dependent on its own gene products or other components (e.g., proteases, reactive oxygen species [ROS], oxidized lipids, unfolded proteins, etc.), regardless of whether such pro-death functions arose through a selection process during evolution (implying a purposeful death) and regardless of whether they wholly or partly cause cell death. For simplicity, we refer to this as “gene-dependent cell death,” without any expectations or requirements that the process would be programmed or regulated (Fig. 1B and 1C). Death-promoting factors can be either direct executors of death (e.g., mammalian caspase-3) or indirect contributors to death (e.g., death signaling pathways). However, applying the term “gene-dependent death” to fungi still requires further considerations.

## CAN WE EXTRAPOLATE FROM ANIMAL KNOCKOUT DATA TO FUNGAL KNOCKOUT DATA?

The evidence suggesting that yeast undergo gene-dependent cell death (PCD, RCD) relies heavily on genetic approaches and the analysis of mutants that are resistant or sensitive to death stimuli. The same powerful genetic approach was used in the landmark C. elegans studies that defined the apoptosis pathway (19), but there is a critical difference. In sharp contrast to yeast, the worm cell death is physiologically relevant by definition—it occurs during normal development. Analogous physiological model systems are limited in availability for the laboratory workhorse Saccharomyces cerevisiae, though there are reports of efforts in this direction (population dynamics, failed mating, quorum sensing, virus infection, sporulation, aging, colony differentiation, and many others) (44–51).

This limitation raises a looming question. Is it reasonable to conclude that a gene has pro-death activity if deletion of that gene confers death resistance to the cell, or could there be unrelated explanations? This is a troublesome question. In fact, researchers in the larger (non-cell death) yeast genetics field are adamant about this issue in my personal experience, and they have an excellent point worthy of consideration. They argue that the improved survival is likely explained by reasons other than the loss of a death-promoting gene. Consider the death-resistant strains of S. cerevisiae lacking either *YCA1*/*MCA1* (metacaspase related to mammalian caspases) or *DNM1*/*DRP-1* (conserved dynamin-like mitochondrial fission factor implicated as an accessory to mammalian cell death) (52–54). In both cases, enzymatic activity is required for the death function. However, if these knockout strains also have heightened defenses or stress responses simply as a consequence of their gene deficiencies, this could potentially account for their ability to survive stress better than the wild type. The same applies to metazoans.

This criticism is difficult to overcome, and yet it has its own caveats. Testing all known adaptive stress responses cannot resolve the criticism; there could always be another untested compensatory mechanism. Conversely, even if adaptive stress responses are elevated, and even if these adaptations partly contribute to death resistance, it remains possible that the gene in question had evolved in part to inflict self-harm. Thus, the possibility of the existence of a cell death mechanism cannot be dismissed because stress responses are heightened, just as a cell death mechanism cannot be inferred from the absence of detectable stress responses. Adaptive responses and pro-death functions are not mutually exclusive possibilities and can even be expected to co-occur. For example, careful analyses revealed that depletion of mammalian apoptosis regulators alter antioxidant defenses at the steady state (55).

Other potential confounders for studying fungal cell death include the secondary mutations affecting cell death that frequently arise in gene knockout strains (56) and the day job functions of most, if not all, pro-death factors that could also significantly impact susceptibility to stress (57–59). Our studies of animal caspases and other pro-death factors in the nervous system revealed alternative and nonapoptotic roles (60–62). Antiapoptotic proteins also have day jobs. How the effects of antiapoptotic mammalian BCL-xL on mitochondria and cellular energetics in healthy cells (57, 58, 63) are related to its antiapoptotic role (BH3/BAX-inhibition) is not yet known. How are we to address all these issues for fungi?

## WHAT IS ACCEPTABLE EVIDENCE OF PROGRAMMED FUNGAL CELL DEATH?

Cell death mechanisms could potentially have evolved in single-cell species to enable them to respond to inevitable pathogens (47) or to live in the differentiated communities of a simple colony harboring layers with distinct transcriptional profiles and death-susceptible subpopulations that likely benefit the community (50, 51, 64). However, except for a few salient examples supporting the existence of fungal cell death pathways (below), the evidence is limited thus far. Based on the history of the metazoan PCD field, the idea of the existence of PCD mechanisms in fungi will remain controversial and lack general acceptance until molecularly defined fungal death pathways are compellingly demonstrated. This includes identification of the direct effectors of fungal cell death and the biochemical mechanisms involved. Until then, claims that fungi undergo gene-dependent PCD/RCD may be overstated without further clarification. Admittedly, the bar is higher now than it was for mammalian PCD in the mid-20th century.

## REASONS TO KEEP OR ABANDON THE TERM “APOPTOSIS” FOR FUNGI

The application of the term "apoptosis" to fungi is difficult to defend by any definition and has been decisively rejected (2, 65–67), and yet studies on “fungal apoptosis” continue to populate the literature (40, 68, 69). This may be an extension of the early metazoan and nonmetazoan literature that was published when "apoptosis" was the only relevant word available and served as a blanket term to describe any type of regulated cell death. However, this practice has declined in recent years to separate apoptosis from several other molecularly defined nonapoptotic death pathways in metazoans (notably necroptosis, ferroptosis, and pyroptosis).

Thus, apoptosis is now more narrowly defined, demanding consideration of authors’ intentions before citing their earlier works as evidence of “apoptosis” as currently defined. The current working definition of mammalian “apoptosis” is caspase-3-mediated cell death (39), and yet fungi do not encode true caspases. Therefore, "apoptosis" applied to fungi presumably has a different definition. Although a defensible consensus definition has not emerged, several criteria have been reported (40). Related fungal metacaspases appear not to behave like mammalian caspases or to be regulated like caspases (70–72). Thus, the use of promiscuous mammalian caspase reporters (e.g., FITC-VAD-FMK) as evidence of “caspase-like” activities in fungi currently lacks rigorous justification (73), and newer better reagents may be helpful (74). However, these facts do not in any way rule out a role for fungal metacaspases or other proteases in cell death, and the possibility remains open.

Is the term “yeast apoptosis” justifiable in other ways? Some justify the use of the term "apoptosis" (or "apoptosis-like") by restricting its use to morphological and biophysical features of dying fungal cells without further implications. Indeed, the word "apoptosis" was put forth in a landmark paper in 1972 (75) without any direct evidence for programmed cell death, supported only by the distinct morphological features of occasional cells in normal human tissues and in rat liver at delayed times after ischemic injury, though the claims have not been free of controversy (76). However, unlike fungi, the apoptotic features of mammalian cells are now recognized as the handiwork of caspase-3, including chromatin condensation, membrane blebbing, and phosphatidylserine exposure on the cell surface to facilitate engulfment into phagocytic cells (where some cells may finish dying) (77–80). Accordingly, the field has advanced. Importantly, apoptosis-like features of mammalian cells not caused by caspases are no longer classified as a type of apoptosis in the latest NCCD guidelines (39). New biochemical or molecular validations may eventually justify the use of the term “apoptosis-like” for fungal death, though it is not presently clear if apoptosis-like fungal death is more like apoptosis than like any other known death mechanism. Perhaps the best justification of the use of the term "fungal apoptosis" is according to the original definition of apoptosis—for use not as a morphological term but to conceptualize the idea of a deliberate cell-autonomous death mechanism. However, an explicit explanation of this otherwise retired definition will be required to avoid the inevitable misunderstandings that can compromise a field’s credibility.

## CONSERVED DEATH PROGRAMS IN FUNGI AND HUMANS?

The new focus is on mammalian cell death by programmed necrosis resulting in plasma membrane rupture. One potentially conserved necrotic cell death mechanism is represented by the proposed N-terminal pore-forming domain of mammalian MLKL, mediator of necroptosis (81). The HET or HeLo-like (HELL) domains of filamentous fungi that mediate cell death upon fusion of two incompatible cells (heterokaryon incompatibility) (82, 83) have an MLKL-like structure prediction (e.g., Phyre2). A different form of death occurs in conidia of the agriculturally important plant pathogen Magnaporthe oryzae (rice blast) during germination (84). Deletion of any of 16 conserved autophagy genes causes conidia to remain alive, blocking both appressorium formation and pathogenicity (85), raising the possibility of autophagy-dependent cell death (ADCD) (86, 87). However, more studies are needed to rule out indirect effects of autophagy (not true ADCD), for example, by degrading an inhibitor of the primary death effector (39). One study suggested that fungi may also undergo iron-dependent ferroptosis, which is thought to result from loss of membrane integrity as a consequence of lipid peroxidation (88), conceivably shared across many species.

Fungi may also encode inhibitors of programmed cell death. Conserved BIR (baculovirus inhibitor of apoptosis repeat) domains that potently suppress cell death were first identified in insect virus genomes and later in humans and *Drosophila* (89). Interestingly, BIR-containing proteins of the plant-pathogenic fungus Botrytis cinerea and the human-pathogenic fungus Aspergillus fumigatus were found to suppress fungal cell death and increase virulence (68, 90). Although the mechanisms are not known, the suggestion that BIR1 suppresses caspase-dependent apoptosis-like fungal cell death is debatable (73), and with rare exceptions, BIR-containing proteins are not direct caspase inhibitors (72, 91). Continuing to stretch mammalian cell death nomenclature to accommodate fungi requires changing ingrained assumptions, and the field may be better served by new nomenclature to convey new discoveries.
